# Challenges in Identifying and Interpreting Intercostal Branches of the Adamkiewicz Artery

**DOI:** 10.3400/avd.cr.25-00092

**Published:** 2025-12-11

**Authors:** Satoru Tomita, Yoshimasa Seike, Tatsuya Nishii, Kazufumi Yoshida, Yojiro Koda, Takayuki Shijo, Yosuke Inoue, Tetsuya Fukuda, Hitoshi Matsuda

**Affiliations:** 1 Departments of Cardiovascular Surgery, National Cerebral and Cardiovascular Center, Suita, Osaka, Japan; 2 Department of Radiology, National Cerebral and Cardiovascular Center, Suita, Osaka, Japan

**Keywords:** thoracic endovascular aortic repair, intercostal artery branching of the Adamkiewicz’s artery, computed tomography angiography

## Abstract

An 82-year-old woman underwent zone 4 thoracic endovascular aortic repair (TEVAR) for a descending aortic aneurysm. Four years later, an additional TEVAR was performed for a type Ib endoleak. Preoperative computed tomography angiography (CTA) initially identified the intercostal artery branching of the Adamkiewicz artery (ICA-AKA) at the left 10th thoracic level, which was covered by a stent graft. Prior to the second TEVAR, CTA showed the ICA-AKA via the left first lumbar artery. Reevaluation of the ICA-AKA is important, particularly after coverage. Surgeons should interpret CTA findings carefully, as other arteries or veins may resemble the AKA.

## Introduction

Spinal cord ischemia (SCI) is one of the most serious complications associated with descending thoracic endovascular aortic repair (TEVAR) and has been reported to have an incidence of 3%–5% during TEVAR.^1)^ Identification of the intercostal or lumbar artery branching of the Adamkiewicz artery (ICA-AKA or LA-AKA) by preoperative imaging and preservation or reconstruction of the ICA-AKA is important for preventing SCI.^2,3)^ The AKA is the largest anterior radiculomedullary artery (RMA) and serves as a major feeder to the anterior spinal artery. However, recent anatomical studies have emphasized that spinal cord perfusion depends not only on the AKA itself but also on multiple RMAs and a collateral vascular network that collectively maintain spinal cord blood flow.^4)^

Magnetic resonance angiography (MRA) and computed tomography angiography (CTA) are used to image and identify the ICA-AKA.^5)^ CTA has the advantage of 3-dimensional visualization, including that of the collateral vessels.^6)^ Here, we present a case in which different ICA-AKAs were identified prior to 2 TEVARs, and we discuss the challenges associated with the identification and interpretation of these vessels.

## Case Report

**Fig. 1:**
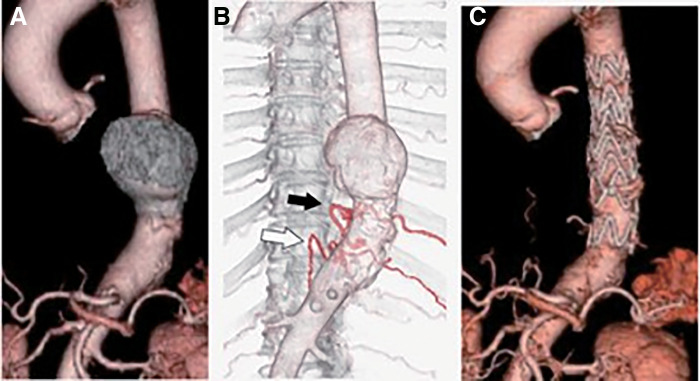
Contrast-enhanced CT findings related to the initial TEVAR. (**A**) Preoperative contrast-enhanced CT revealed a 50-mm saccular aneurysm in the descending aorta. (**B**) Preoperative image of the ICA-AKA, revealing a left 10th intercostal artery origin (black arrow). The left 11th ICA, which is a collateral artery to the ICA-AKA, was detected (white arrow). (**C**) The identified ICA-AKA is covered with a stent graft. TEVAR: thoracic endovascular aortic repair; ICA-AKA: intercostal artery branching of the Adamkiewicz artery; CT: computed tomography

An 82-year-old female with a history of hypertension, dyslipidemia, Parkinson’s disease, and osteoporosis was referred for treatment of a degenerative descending saccular aneurysm (50 mm) (**Fig. 1A**). Preoperative CTA revealed that the AKA branched from the left 10th intercostal artery (**Fig. 1B**), which was included in the distal landing zone of the TEVAR.

The initial TEVAR was performed with 2 stent grafts, RelayPro (28N4-26-104-26X, 28N4-30-104-30X) (Bolton Medical, Sunrise, FL, USA), placed sequentially from the proximal side (Th5) to the distal side (Th11). A postoperative CTA revealed no endoleaks (**Fig. 1C**).

The ICA-AKA was covered with a stent graft, and there was no change in the intraoperative motor-evoked potential (MEP). The postoperative course was uneventful. A cerebrospinal fluid drainage (CSFD) tube was inserted prophylactically before TEVAR; however, because there were no symptoms of SCI, it was removed on the second day after TEVAR without being used.

**Fig. 2:**
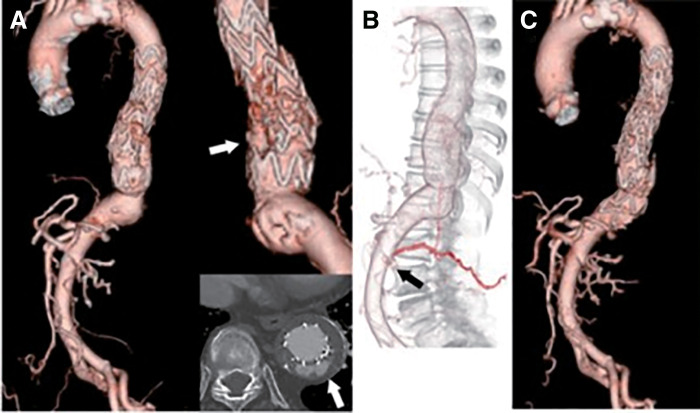
Contrast-enhanced CT findings related to the additional TEVAR. (**A**) The descending aortic aneurysm is re-enlarged to 54 mm with a type Ib endoleak (white arrow). (**B**) The ICA-AKA, originating from the left L1 lumbar artery (black arrow), differs from the previously identified ICA-AKA. (**C**) An additional zone 4 TEVAR was performed to extend the distal landing zone without covering the newly identified ICA-AKA. TEVAR: thoracic endovascular aortic repair; ICA-AKA: intercostal artery branching of the Adamkiewicz artery; CT: computed tomography

During follow-up, the descending aortic aneurysm shrank to 44 mm over 2 years. However, follow-up CTA revealed sac reenlargement to 54 mm with a type Ib endoleak 4 years after TEVAR (**Fig. 2A**); therefore, an additional TEVAR was planned. Because the ICA-AKA had been closed during the previous TEVAR procedure, CTA was used to reevaluate the blood flow to the AKA. CTA showed that the left first lumbar artery branched from a different AKA compared to the previous TEVAR procedure (**Fig. 2B**). TEVAR was performed to extend the distal landing zone without covering the newly identified ICA-AKA using a Zenith Alpha (ZTA-P-28-155-W1) (Cook Medical, Bloomington, IN, USA). There was no significant change in the MEP without prophylactic CSFD tube insertion, and the postoperative course was uneventful, with no endoleaks on CTA (**Fig. 2C**). The aneurysm diameter shrank and remained stable for 2 years after the second TEVAR procedure.

## Discussion

**Fig. 3:**
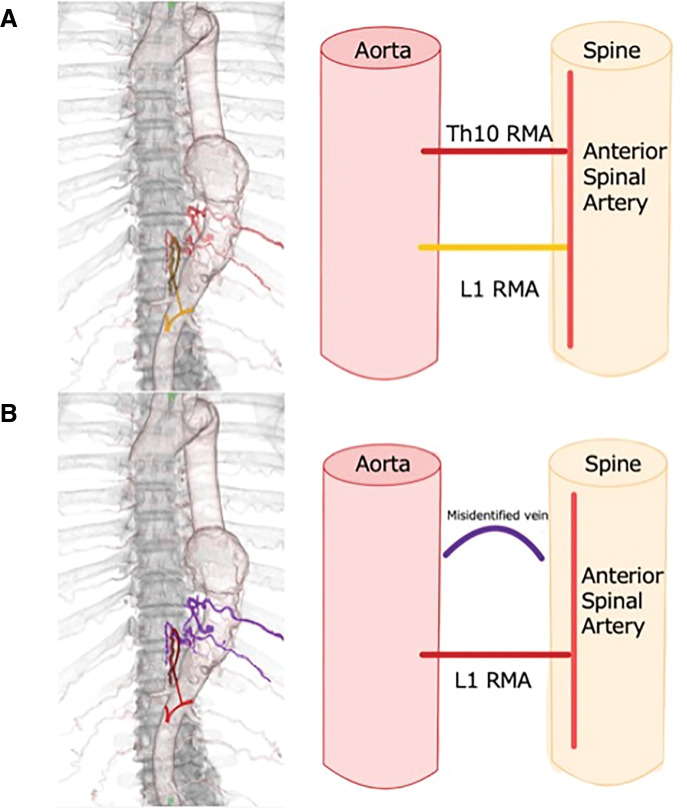
Schematic illustrations of 2 hypotheses explaining the discrepancy in the identification of the ICA-AKA between the first and second TEVAR procedures. (**A**) Two RMAs contributed to spinal cord perfusion. The RMA, diagnosed as the AKA, originated from the left 10th intercostal artery and was covered by a stent graft during the initial TEVAR. Subsequently, a previously existing radiculomedullary artery (L1 RMA) originating from the left first lumbar artery was more clearly visualized. (**B**) The vessel initially identified at the left 10th intercostal level was likely a venous structure mimicking the hairpin configuration of the AKA, and the RMA that should have been identified as the AKA was actually derived from the left first lumbar artery. TEVAR: thoracic endovascular aortic repair; ICA-AKA: intercostal artery branching of the Adamkiewicz artery; RMAs: radiculomedullary arteries

In this case, the discrepancy in AKA identification before the 2 TEVARs can be attributed to 2 factors. First, the 2 primary RMAs may have contributed to spinal cord blood supply from the beginning (**Fig. 3A**). Second, the CTA findings may have been misinterpreted (**Fig. 3B**).

First, among the 2 primary RMAs, only the dominant one was initially identified. However, once the dominant vessel was occluded by a stent graft, the non-dominant RMA developed further and became visible. It is important to note that TEVAR causes hemodynamic alterations and can lead to the development of alternate pathways to the spinal cord. Alvernia et al. previously reported that segmental RMAs, which supply blood to the thoracic and lumbar spinal cords, may be unilateral, bilateral, or multiple.^4)^ In this anatomical study of the AKA and other RMAs, 32% of the cadaver specimens had an RMA equivalent to the AKA but not the AKA itself. These findings suggest a possible shift in the conventional concept of dependence on a single RMA (i.e., AKA), highlighting the difficulty of the current diagnosis and emphasizing the importance of a collateral circulation network.

The second reason involves a recognized limitation in the CTA interpretation, specifically, the difficulty in distinguishing the characteristic hairpin curve of the anterior radicular artery that characterizes the AKA from similar venous structures. The diameter of the ICA-AKA typically ranges from 0.5 to 1.5 mm, and accurate delineation can be challenging due to factors such as contrast timing and imaging conditions. The detection rates of AKA using CTA have been reported to be 80%–90%.^7,8)^ Accurate identification of the hairpin curve and verification of the vessel continuity through the intervertebral foramen and into the aorta are crucial. In addition, the washout phenomenon in the venous phase can help distinguish between arteries and veins.^9)^

In the present case, continuity of the vascular structure within the intervertebral foramen could not be definitively observed on the initial preoperative CT scan. This raises the possibility that the vein was misidentified as the anterior radicular artery. However, the presence of washout during the venous phase did not confirm that the vessel was a vein, even after retrospective analysis by radiologists. Surgeons should exercise caution when interpreting AKA reports from CTA because different CTA techniques and contrast timings can produce inconsistent results. Therefore, although challenges remain in identifying AKA by CT, reevaluating the spinal cord blood supply pathways is an effective way to confirm the presence of other or new collateral vessels for additional TEVAR after the ICA-AKA is occluded in previous TEVAR procedures, as in the present case.

## Conclusion

We encountered cases in which the ICA-AKA was identified at different sites prior to 2 TEVAR procedures performed at separate time periods. Surgeons must exercise caution when interpreting imaging findings of the AKA, as other MRAs resemble the AKA, and veins may be misdiagnosed as the AKA.
